# Primary Hepatic Angiosarcoma: Distinct Imaging Phenotypes Mirroring Histopathologic Growth Patterns in a Retrospective Human Study

**DOI:** 10.3390/diagnostics16020291

**Published:** 2026-01-16

**Authors:** Byoung Je Kim, Jung Hee Hong, Hye Won Lee

**Affiliations:** 1 Department of Radiology, Keimyung University Dongsan Hospital, Keimyung University School of Medicine, 1035, Dalgubeol-daero, Dalseo-gu, Daegu 42601, Republic of Korea; keke4@naver.com; 2 Department of Pathology, Keimyung University Dongsan Hospital, Keimyung University School of Medicine, 1035, Dalgubeol-daero, Dalseo-gu, Daegu 42601, Republic of Korea; hwlee@dsmc.or.kr

**Keywords:** primary hepatic angiosarcoma, radiologic–pathologic correlation, liver neoplasm, computed tomography, magnetic resonance imaging, histologic growth pattern, vasoformative pattern, non-mass-forming lesions, retrospective study

## Abstract

**Background/Objectives:** To date, no studies have examined radiologic findings by histologic patterns of primary hepatic angiosarcoma; this study clarified radiologic findings of primary hepatic angiosarcoma according to distinct histologic patterns. **Methods:** From January 2010 to October 2024, 17 individuals (mean age, 69 years ± 11; 11 men) with pathologically confirmed primary hepatic angiosarcoma underwent computed tomography (CT) with or without magnetic resonance imaging (MRI). Histologic patterns were classified as mass-forming, subdivided into vasoformative and non-vasoformative (epithelioid and spindled) patterns, or non-mass-forming, subdivided into sinusoidal and peliotic patterns. Two radiologists independently reviewed CT and MRI images, classifying lesions as non-mass-forming or mass-forming. Hypervascular portions and targetoid patterns were also assessed. Associations between histologic patterns and radiologic findings were evaluated using Fisher’s exact test. **Results:** Mass-forming tumors were observed in 13 individuals (76.5%), and non-mass-forming tumors in 4 individuals (23.5%). Significant correlation (*p* < 0.05) was found between radiologic classification (non-mass-forming or mass-forming) and corresponding pathologic patterns. Pathologic subdivision into vasoformative and non-vasoformative patterns did not correlate with hypervascular portions on imaging. **Conclusions:** Pathological classification into mass-forming and non-mass-forming patterns corresponds closely to radiologic classification of mass-forming and non-mass-forming lesions, indicative of strong pathologic features in imaging.

## 1. Introduction

Hepatic angiosarcoma is the most common malignant mesenchymal tumor of the liver but accounts for <2% of primary hepatic neoplasms, making it a rare yet highly aggressive malignancy. Arising from endothelial cells, hepatic angiosarcoma progresses rapidly and is associated with a poor prognosis; most untreated individuals die within 6 months [1], and most who undergo surgical resection die within 1 year [2,3]. Although environmental carcinogens such as Thorotrast, vinyl chloride, and arsenic have been historically linked to its development, many recent cases show no known exposure, indicating an incomplete understanding of its etiology [1,4].

Hepatic angiosarcoma typically consists of a highly vascular tumor composed of poorly organized vascular channels, substantially increasing the risk of hemorrhage during diagnostic percutaneous biopsy [5,6,7]. Although cross-sectional imaging provides a noninvasive diagnostic option, previous studies have shown that imaging characteristics of primary hepatic angiosarcoma often overlap with those of metastases, hemangiomas, and hepatocellular carcinoma [1,8,9,10,11,12,13,14,15,16,17]. Therefore, pathological confirmation remains essential for establishing a definitive diagnosis. However, previous imaging studies have reported inconsistent correlations between radiologic patterns and histopathologic subtypes, with some describing overlapping enhancement features among vasoformative and non-vasoformative lesions. These discrepancies highlight the ongoing uncertainty surrounding the imaging–pathologic interpretation of primary hepatic angiosarcoma.

A pivotal pathological study by Yasir and Torbenson introduced a morphological classification of hepatic angiosarcoma [18], offering new insights into diagnostic interpretation. Building on that framework, this study examines whether these histopathologic patterns are reflected in cross-sectional imaging features to improve diagnostic accuracy and deepen clinical understanding of primary hepatic angiosarcoma.

## 2. Materials and Methods

This study was conducted in accordance with the Declaration of Helsinki and approved by the Institutional Review Board of Keimyung University Dongsan Hospital, with the requirement for informed consent waived due to the retrospective nature of data collection (protocol code 17 November 2024 and date of approval 12 November 2024).

### 2.1. Patient and Clinical Data Collection

**Figure 1:**
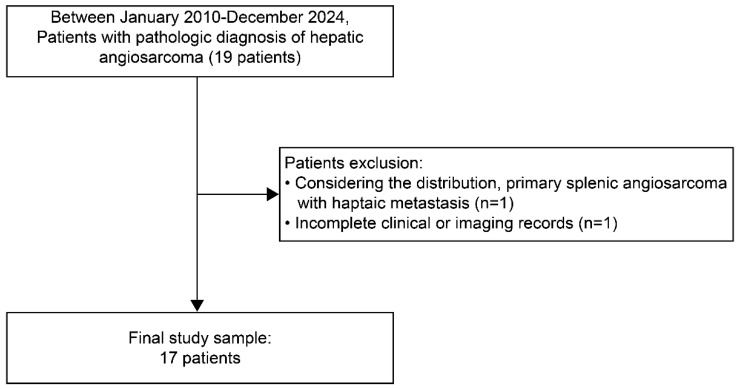
Flow diagram of the patient inclusion procedure.

A comprehensive search of electronic health records (BESTCare version 2.0, ezCaretech) was conducted between October 2010 and December 2024. Nineteen individuals with pathologically confirmed hepatic angiosarcoma were initially identified (Figure 1). Inclusion criteria were: (1) a pathological diagnosis of primary hepatic angiosarcoma and (2) availability of cross-sectional imaging within 3 months of histologic confirmation.

Individuals were excluded if they (1) had evidence of angiosarcoma originating from an extrahepatic site with secondary hepatic metastasis or (2) lacked accessible cross-sectional imaging within 3 months of diagnosis.

Clinical data were retrospectively obtained from institutional databases, including demographic variables (age, sex) and laboratory values such as aspartate aminotransferase, alanine aminotransferase, alkaline phosphatase, total bilirubin, international normalized ratio (INR), albumin, platelet count, carbohydrate antigen 19-9 (CA 19-9), alpha-fetoprotein (AFP), hepatitis B surface antigen, and anti-hepatitis C virus antibody.

### 2.2. Computed Tomography (CT) and Magnetic Resonance Imaging (MRI) Examinations

CT examinations were performed on 64- or 128-detector row scanners (Somatom Definition AS, Somatom Definition Edge; Siemens Healthcare, Forchheim, Germany). Protocols included non-enhanced, arterial phase, and portal venous phase acquisitions, with 3–5 mm slice thicknesses. Non-ionic iodinated contrast material was administered intravenously using a power injector at approximately 2 mL/kg (maximum 150 mL) at a flow rate of 3 mL/s. Arterial phase images were obtained 10–15 s after the aortic attenuation reached 100 Hounsfield units, and portal venous phase images were acquired after a fixed 75 s delay. Scans were obtained at 100–120 kVp and reconstructed using filtered back projection.

MRI was performed using 1.5- or 3-T scanners (Magnetom Avanto, Magnetom Vida; Siemens Healthcare). Protocols included axial dual-echo T1-weighted in-phase and out-of-phase imaging, half-Fourier single-shot turbo spin-echo T2-weighted imaging in axial and coronal planes, respiratory-triggered diffusion-weighted imaging (b values: 0, 50, 500, 900 s/mm^2^), and dynamic contrast-enhanced T1-weighted imaging before and after administration of gadoxetic acid (Primovist; Bayer Schering Pharma, Berlin, Germany). Contrast material was injected at 0.025 mmol/kg at 1 mL/s, followed by a 10 mL saline flush. Arterial, portal venous, transitional, and hepatobiliary phase images were obtained at 5 s after peak enhancement, 50 s, 3 min, and 20 min, respectively.

### 2.3. Pathological and Imaging Analysis

Based on established pathological criteria [18,19], an experienced liver pathologist (H.W.L., with over 10 years of diagnostic expertise) categorized tumors into mass-forming and non-mass-forming patterns. The mass-forming group was further subclassified into vasoformative and non-vasoformative types; non-vasoformative types comprised epithelioid and spindle cell morphologies. The non-mass-forming group included sinusoidal and peliotic growth patterns.

For radiologic classification designed to parallel these histologic patterns, one radiologist (B.K., 8 years of experience in abdominal imaging) applied a predefined imaging categorization scheme to classify lesions as mass-forming or non-mass-forming. Mass-forming lesions were evaluated for hypervascularity, and non-mass-forming lesions were assessed for infiltrative or peliotic features.

To evaluate the imaging findings in a blinded and reproducible manner, two radiologists (B.K. and J.H.H., each with >6 years of abdominal imaging experience), blinded to clinical and pathological information, independently reviewed all imaging studies. Any discrepancy was resolved by consensus among the radiologists.

### 2.4. Statistical Analysis

Associations between histologic patterns and imaging features were evaluated using Fisher’s exact test. Interobserver agreement between radiologists was calculated using Cohen’s kappa statistics and interpreted as follows: poor (<0.20), fair (0.21–0.40), moderate (0.41–0.60), good (0.61–0.80), and excellent (0.81–1.00).

Statistical analyses were performed using SPSS software (version 21.0; IBM Corp., Armonk, NY, USA).

## 3. Results

### 3.1. Patient Characteristics

Among the 19 individuals initially screened, two were excluded: one due to splenic angiosarcoma with hepatic metastasis and the other due to the absence of cross-sectional imaging within 3 months of pathological diagnosis. Seventeen individuals with pathologically confirmed primary hepatic angiosarcoma were therefore included in the final analysis.

Of these, 11 (64.7%) were male and 6 (35.3%) were female, with a mean age of 69 ± 11 years (Figure 1).

Most individuals demonstrated normal serum AFP and CA 19-9 levels, indicating limited diagnostic value of these tumor markers for hepatic angiosarcoma. Thrombocytopenia was observed in several individuals, potentially reflecting portal hypertension or hypersplenism. Liver enzymes showed variable elevations, and INR prolongation was identified in a subset of individuals, suggesting impaired hepatic synthetic function. Detailed demographic and clinical data are provided in Table 1.

### 3.2. Pathological Results

Among the 17 individuals, 16 were diagnosed based on core needle biopsy specimens, and 1 underwent surgical resection.

Histologically, 13 individuals (76.5%) displayed a mass-forming pattern, whereas 4 individuals (23.5%) demonstrated a non-mass-forming pattern.

Within the mass-forming group, eight individuals exhibited a vasoformative pattern and five demonstrated a non-vasoformative pattern.

Among the non-vasoformative subtypes, three were classified as epithelioid type and two as spindle cell type.

**Figure 2:**
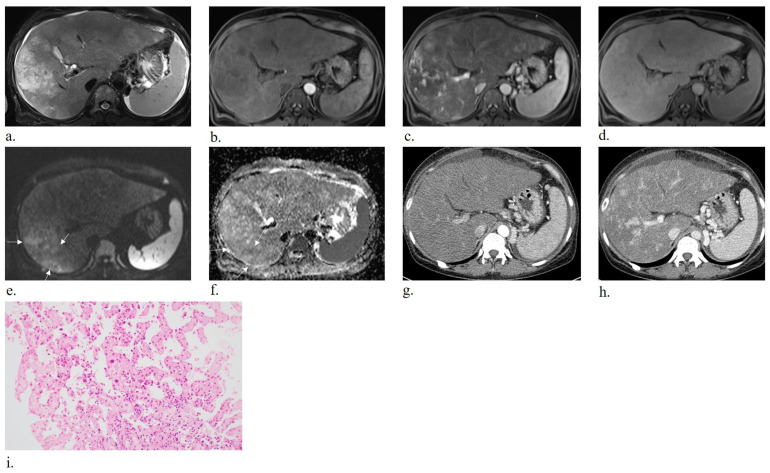
Fifty-seven-year-old woman with chronic hepatitis B diagnosed with primary hepatic angiosarcoma, presenting as non-mass-forming lesions with a sinusoidal growth pattern. Axial T2-weighted MRI (**a**) shows ill-defined, hyperintense lesions involving both hepatic lobes with hepatomegaly. Pre-contrast T1-weighted imaging (**b**) reveals hypointensity. In the arterial phase (**c**), peripheral hyperenhancement is seen. In the hepatobiliary phase (**d**), the margins are poorly defined with iso- to hypointensity. Diffusion-weighted imaging and the ADC map ((**e**,**f**); b = 800 s/mm^2^) show areas of diffusion restriction (arrows). Arterial-phase (**g**) and portal venous phase (**h**) CT images depict ill-defined lesions with heterogeneous parenchymal enhancement and hepatomegaly. Histopathology (**i**) with hematoxylin-eosin staining demonstrates cytologic atypia of endothelial cells lining dilated sinusoidal spaces among residual hepatic trabeculae.

**Figure 3:**
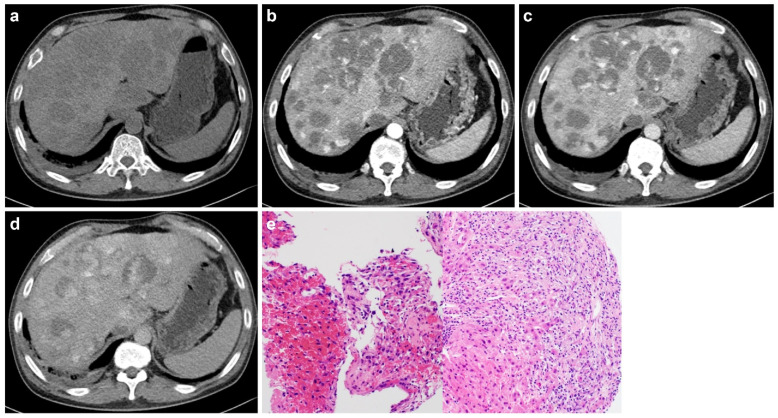
Sixty-one-year-old woman with no significant medical history diagnosed with primary hepatic angiosarcoma exhibiting a non-mass-forming peliotic growth pattern. Unenhanced computed tomography (CT; (**a**)) shows multiple low-attenuation lesions. Dynamic contrast-enhanced CT in the arterial (**b**), portal venous (**c**), and delayed phases (**d**) shows progressive centripetal and peripheral enhancement. Systemic bone metastases were present but are not shown. Histopathology (**e**) with hematoxylin-eosin staining demonstrates oval-shaped tumor cells infiltrating in an alveolar-like arrangement within blood-filled spaces.

**Figure 4:**
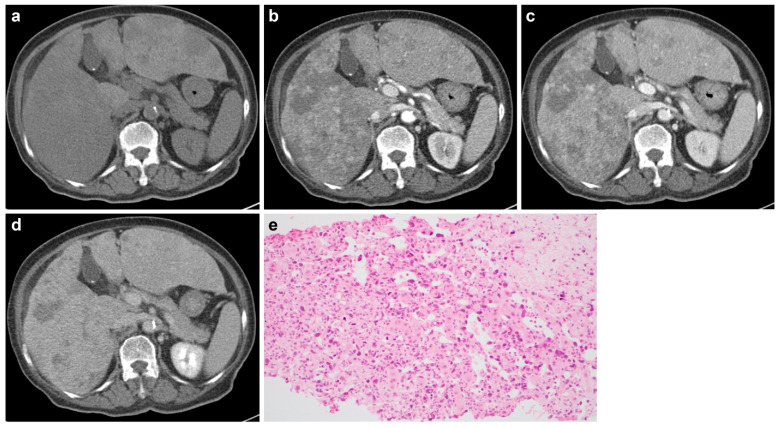
Eighty-five-year-old woman with hypertension and diabetes diagnosed with mass-forming, vasoformative primary hepatic angiosarcoma. Unenhanced CT (**a**) demonstrates multiple low-density masses involving both lobes with hepatomegaly. Arterial (**b**) and portal venous (**c**) phases show nodular or irregular flame-shaped enhancement. The delayed phase (**d**) reveals progressive yet incomplete enhancement. These findings are consistent with the classic imaging features of hepatic angiosarcoma described in the literature. Histopathology (**e**) with hematoxylin-eosin staining reveals a vasoformative pattern with irregular proliferation of abnormal vascular structures admixed with pleomorphic tumor cells.

**Figure 5:**
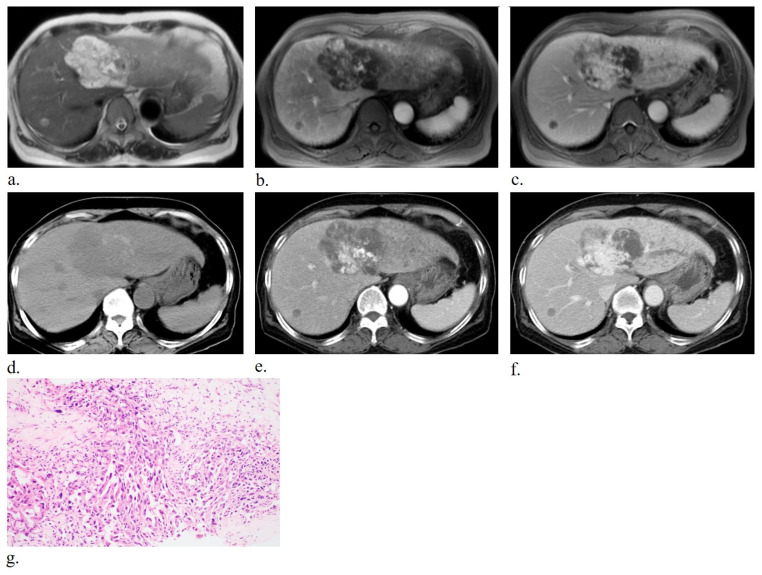
Seventy-two-year-old woman without prior medical history diagnosed with mass-forming primary hepatic angiosarcoma displaying a spindle cell morphology predominating over the epithelioid component and demonstrating a non-vasoformative pattern. T2-weighted magnetic resonance imaging (MRI; (**a**)) shows a well-defined lesion with bright high signal in the left lobe and small T2 intermediate-to-high signal nodule in the right lobe, suggestive of intrahepatic metastasis. On arterial-phase T1-weighted MRI (**b**), the lesion appears irregular with central hyperenhancement. The portal venous phase (**c**) shows peripheral enhancement progression. Non-contrast computed tomography (CT; (**d**)) shows internal calcifications. Arterial (**e**) and portal (**f**) phases of contrast-enhanced CT show central enhancement similar to the aorta with peripheral progression of contrast filling. Histopathology (**g**) with hematoxylin-eosin staining shows atypical endothelial cells lining dilated sinusoidal spaces between hepatic trabeculae.

In the non-mass-forming group, three individuals showed a sinusoidal growth pattern and one demonstrated a peliotic pattern (Figure 2, Figure 3, Figure 4 and Figure 5).

### 3.3. Comparison of Histological Pattern and Radiological Classification

A statistically significant association was observed between histologically determined growth patterns (mass-forming vs. non-mass-forming) and their corresponding imaging classifications (Fisher’s exact test, two-sided *p* = 0.035, <0.05).

However, within the mass-forming group, no significant correlation was found between vasoformative vs. non-vasoformative histologic patterns and presence or absence of hypervascularity on imaging.

The minimal dataset supporting the findings of this study is provided in the Supplementary Materials.

### 3.4. Interobserver Agreement

Interobserver agreement between the two radiologists for classifying tumors as mass-forming or non-mass-forming was substantial (κ = 0.78).

Agreement for evaluating hypervascularity in mass-forming tumors was moderate (κ = 0.61), while agreement for identifying infiltrative or peliotic characteristics in non-mass-forming tumors was substantial (κ = 0.74).

## 4. Discussion

This study demonstrates that the imaging manifestations of primary hepatic angiosarcoma (PHA) closely reflect the underlying histopathologic growth patterns, particularly the distinction between mass-forming and non-mass-forming phenotypes. In this cohort, mass-forming tumors appeared as discrete nodular or mass-like enhancing lesions on CT and MRI, whereas non-mass-forming tumors presented as diffuse parenchymal infiltration without a definable mass. These imaging characteristics corresponded to established histologic subtypes, including vasoformative, epithelioid, spindle cell, sinusoidal, and peliotic patterns.

Aligned with the pathological framework proposed by Yasir and Torbenson, PHA was broadly categorized into mass-forming types defined by vascular channel formation or solid cellular proliferation and non-mass-forming types characterized by sinusoidal or peliotic growth [18]. Our results indicated that radiologic differentiation between these phenotypes is achievable: mass-forming tumors commonly demonstrated heterogeneous arterial enhancement, often with irregular or flame-like morphology, followed by progressive yet incomplete enhancement during the portal venous and later phases. Conversely, non-mass-forming tumors showed ill-defined infiltrative enhancement with patchy or mosaic parenchymal alterations and scattered foci of arterial uptake, consistent with sinusoidal tumor spread.

Our study did not identify a direct association between the radiologic degree of hypervascularity and underlying histologic subtype (vasoformative vs. non-vasoformative), possibly because cross-sectional imaging cannot fully depict the microscopic vascular architecture or because of heterogeneity within tumor subtypes. Both vasoformative and non-vasoformative patterns of hepatic angiosarcoma demonstrated prominent peripheral hypervascularity on contrast-enhanced CT. This observation likely reflects the inherently high vascularity of the tumor regardless of subtype. In vasoformative tumors, irregular and dilated vascular channels lined by malignant endothelium may produce increased peripheral perfusion, resulting in intense rim enhancement on imaging [20]. In non-vasoformative tumors, although well-formed luminal structures are absent histologically, abundant microvasculature generated through angiogenesis may concentrate near the lesion margins, also contributing to marked peripheral enhancement [20]. Thus, whether the tumor forms explicit vascular channels or grows in a solid pattern, the periphery appears to remain highly vascularized, which may explain the characteristic hyperenhancing rim commonly observed on contrast-enhanced CT imaging.

The correlation between imaging and histology carries important clinical implications. As biopsy of highly vascular tumors carries a substantial hemorrhagic risk, improved radiologic recognition of PHA may reduce the need for invasive procedures in selected situations. In particular, familiarity with characteristic imaging features, including multifocal hepatic lesions with hemorrhagic components, progressive enhancement without definitive washout, and the absence of major vascular invasion, raises suspicion for PHA even before histologic confirmation.

Despite these recognizable imaging traits, differentiating PHA from other vascular lesions remains challenging. Hepatic hemangiomas resembled PHA by demonstrating progressive peripheral enhancement [21]; however, classic hemangiomas typically show smooth, nodular, discontinuous enhancement with complete centripetal fill-in, in contrast to the irregular and incomplete enhancement seen in PHA [22]. Additionally, PHA often contains hemorrhagic elements, heterogeneous internal structure, and multifocal involvement features that are uncommon in benign hemangiomas [10].

Several factors assist in differentiating PHA from HCC. PHA typically arises in a non-cirrhotic liver and does not elevate AFP, whereas HCC often develops in cirrhosis and shows increased AFP levels [23]. On imaging, HCC generally demonstrates early arterial enhancement with rapid washout in the portal or venous phase [15]. HCC also commonly exhibits portal vein invasion, whereas this finding is uncommon in PHA [24]. When hepatic lesions occur without cirrhosis, washout, vascular invasion, or elevated AFP, PHA should be considered as an alternative diagnosis [23].

Hypervascular liver metastases, including those from neuroendocrine tumors, may resemble PHA when multiple and vascular [10]. However, enhancement kinetics, lesion distribution, and clinical identification of a primary malignancy usually distinguish metastases from PHA [22]. For instance, metastases might display more uniform enhancement or show features of their primary tumor (e.g., a neuroendocrine tumor metastasis might show intense arterial enhancement and particular tumor markers), whereas PHA often demonstrates multifocal hemorrhagic lesions, persistent enhancement without classic washout, and no extrahepatic primary tumor.

Rare entities such as peliosis hepatis and epithelioid hemangioendothelioma (EHE) can resemble hepatic angiosarcoma on imaging. Peliosis hepatis contains blood-filled cavities and may show delayed enhancement, and EHE can present with multiple nodules and gradual enhancement. However, correlation of imaging findings with clinical context usually allows differentiation from PHA [25,26]. For instance, EHE often demonstrates capsular retraction and tends to be less aggressive, and peliosis is typically associated with specific clinical conditions or medications. These clues, combined with the absence of systemic malignant features, help distinguish these benign or low-grade vascular lesions from aggressive PHA.

Our interobserver agreement analysis supports the robustness of the proposed imaging classification system. There was substantial agreement in categorizing lesions as mass-forming vs. non-mass-forming, indicating that this distinction can be made reproducibly on imaging. This concordance reinforces that the growth pattern, nodular mass versus infiltrative spread, is usually apparent on scans. Agreement on evaluating lesion hypervascularity was moderate, likely reflecting subjectivity in judging enhancement; different observers may apply slightly different thresholds for designating a lesion as hypervascular. Nonetheless, the overall reproducibility of the imaging interpretation was acceptable, suggesting that with clear criteria, radiologists can consistently identify key imaging features of PHA.

Notably, prior case reports have described hepatic angiosarcoma presenting with imaging features that mimicked other hepatic conditions, often leading to misdiagnosis when the broader context was lacking. For instance, some reports described diffuse infiltrative liver lesions in PHA that exhibited cirrhosis-like morphology with features of portal hypertension, leading to initial misdiagnosis of infiltrative HCC [27,28,29,30]. In other cases, PHA lesions were reported to resemble peliosis hepatis due to blood-filled cavities and delayed enhancement, resulting in diagnostic confusion [25]. These reports were isolated and lacked systematic radiologic–pathologic correlation. Our study builds on these observations by demonstrating that these imaging phenotypes correspond to distinct histologic subtypes of PHA. The infiltrative, cirrhosis-mimicking pattern aligns with the sinusoidal growth subtype, whereas cases resembling peliosis hepatis correspond to the peliotic subtype. This structured radiologic–pathologic linkage provides a refined diagnostic framework for PHA. Recognizing these imaging clues considering their histologic basis enables more confident, non-invasive identification of PHA subtypes and improves diagnostic precision. In other words, previously puzzling radiologic mimics can now be understood as manifestations of PHA’s variable pathology.

This study has certain limitations. First, the study cohort was small, reflecting the rarity of primary hepatic angiosarcoma, and was derived from a single institution, introducing potential selection bias. Second, the retrospective study design may limit generalizability. Third, although histopathologic confirmation served as the reference standard, the majority of cases (94%, 16/17) were diagnosed using core needle biopsy rather than surgical resection. Given the marked histologic heterogeneity of hepatic angiosarcoma, biopsy samples may not fully capture the entire spectrum of tumor growth patterns within a lesion. In particular, limited sampling may preferentially reflect a dominant or more accessible component, such as vasoformative or solid areas, while underrepresenting coexisting sinusoidal or peliotic growth patterns. This limitation may partially account for discrepancies between imaging phenotypes and histologic subtypes observed in some cases and should be considered when interpreting radiologic–pathologic correlations.

Fourth, imaging studies were performed within a 3-month interval before or after pathologic diagnosis, and this interval was not uniform across all patients. During this period, tumor progression, intratumoral hemorrhage, or interval-related changes may have altered imaging appearances, potentially influencing the classification of imaging patterns and their correlation with histologic findings. Fifth, CT and MRI findings were analyzed together, and the incremental diagnostic contribution of individual imaging modalities and MRI-specific sequences, such as hepatobiliary phase imaging and diffusion-weighted imaging, was not evaluated separately. This approach was adopted because of the small sample size and heterogeneity in imaging protocols, as not all patients underwent both CT and MRI or had uniform MRI sequences available. Finally, because of the definitional discrepancy between a radiologic mass, defined as a three-dimensional, space-occupying lesion visible on imaging, and a pathologic mass, which may represent abnormal tissue without forming a discrete tumor [31,32], the peliotic growth pattern was categorized as non-mass-forming despite occasionally appearing space-occupying radiologically. This limitation may have influenced classification and warrants consideration in future efforts to establish unified radiologic–pathologic criteria.

Nonetheless, this study highlights the value of integrating imaging features with histologic growth patterns in the noninvasive diagnosis of PHA. Future multicenter, prospective studies with larger cohorts are warranted to validate these findings and refine diagnostic criteria.

Advances in artificial intelligence and machine learning may enhance diagnostic accuracy by enabling automated detection of subtle imaging features. Considering the poor prognosis and hemorrhagic risks associated with PHA [11], improved noninvasive diagnostic tools impacted clinical management, including earlier diagnosis, risk stratification, and therapy monitoring, particularly as targeted therapies and immunotherapies continue to develop.

In conclusion, primary hepatic angiosarcoma demonstrated imaging phenotypes that generally parallel its histopathologic growth patterns. Recognition of these characteristic imaging features—whether a tumor appears mass-forming with heterogeneous vascular channels or diffusely infiltrative with sinusoidal spread—may assist in suggesting the diagnosis of PHA. Improved awareness of these imaging manifestations has the potential to support more informed clinical decision-making, particularly by raising suspicion for this rare and aggressive malignancy and potentially reducing unnecessary delays or invasive procedures.

## Figures and Tables

**Table 1:** Demographic and clinical characteristics of the study patients.

Characteristic	Value
No. of patients	17
Age (years)	69 ± 11 (mean ± SD, n = 17)
Sex (Male/Female)	11/6
AST (U/L)	55.0 ± 39.7 (mean ± SD, n = 17)
ALT (U/L)	31.2 ± 19.9 (mean ± SD, n = 17)
ALP (U/L)	286.8 ± 351.7 (mean ± SD, n = 17)
Total bilirubin (mg/dL)	2.61 ± 3.75 (mean ± SD, n = 17)
INR	1.25 ± 0.17 (mean ± SD, n = 17)
Albumin (g/dL)	3.41 ± 0.58 (mean ± SD, n = 17)
Platelet count (×10^3^/mm^3^)	144,000 (IQR 100,000–219,000; median, n = 17)
CA 19-9 (U/mL)	8.30 (range 1.41–1086.00; median, n = 11)
AFP (ng/mL)	5.96 ± 12.55 (mean ± SD, n = 13)
HBsAg (Positive/Negative)	2/15 (13.3%)
Anti-HCV Ab (Positive/Negative)	0/15 (0%)

Values are expressed as mean ± standard deviation or median (range), as appropriate. AFP, alpha-fetoprotein; ALP, alkaline phosphatase; ALT, alanine aminotransferase; AST, aspartate aminotransferase; CA, carbohydrate antigen; HBsAg, hepatitis B surface antigen; Anti-HCV Ab, anti-hepatitis C virus antibody; IQR, interquartile range.

## Data Availability

The data presented in this study are available on request from the corresponding author due to privacy restrictions associated with patient information.

## Supplementary Materials

The following supporting information can be downloaded at: https://www.mdpi.com/article/10.3390/diagnostics16020291/s1, Table S1: Minimal Dataset Detailing Imaging and Pathologic Findings of Each Patient with Primary Hepatic Angiosarcoma.

## Abbreviations

AFP	Alpha-fetoprotein
ALP	Alkaline phosphatase
ALT	Alanine aminotransferase
AST	Aspartate aminotransferase
CA 19-9	Carbohydrate antigen 19-9
CT	Computed tomography
H&E	Hematoxylin and eosin
HBsAg	Hepatitis B surface antigen
HCC	Hepatocellular carcinoma
INR	International normalized ratio
IQR	Interquartile range
MRI	Magnetic resonance imaging
PHA	Primary hepatic angiosarcoma
ROI	Region of interest
SPSS	Statistical Package for the Social Sciences
