# Supplementary material for: Primary Hepatic Angiosarcoma: Distinct Imaging Phenotypes Mirroring Histopathologic Growth Patterns in a Retrospective Human Study
Source: Diagnostics (Basel). 2026 Jan 16;16(2):291. doi: 10.3390/diagnostics16020291 (PMC12840377; doi:10.3390/diagnostics16020291)
Supplement: Supplementary file 1 [file diagnostics-16-00291-s001.zip › diagnostics-4055937-supplementary.pdf]

Supplementary Table S1. Minimal Dataset Detailing Imaging and Pathologic Findings of Each Patient with Primary Hepatic Angiosarcoma

No.	Sex	Age	Pathologic findings	Imaging findings
1	F	57	Non-mass forming, sinusoidal pattern	Non-mass forming, infiltrative
2	F	85	Mass-forming, vasofromative	Mass-forming, hypervascular
3	M	62	Mss-forming non-vasofrmative, epithelioid	Mass-forming, hypervascular
4	F	77	Non-mass forming, sinusoidal pattern	Mass-forming, no hypervascularity
5	F	82	Mss-forming non-vasofrmative, epithelioid	Mass-forming, hypervascular
6	M	72	Mass-forming, vasofromative	Mass-forming, hypervascular
7	M	61	Non-mass forming, sinusoidal pattern	Mass-forming, hypervascular
8	M	71	Non-mass forming, peliotic pattern	Non-mass forming, periotic
9	M	80	Mss-forming, non-vasofrmative, epithelioid	Mass-forming, no hypervascularity
10	M	64	Mass-forming, vasofromative	Mass-forming, hypervascular
11	F	72	Mss-forming non-vasofrmative, spindled	Mass-forming, hypervascular
12	M	68	Mass-forming, vasofromative	Mass-forming, hypervascular
13	M	80	Mass-forming, vasofromative	Mass-forming, hypervascular
14	M	46	Mss-forming non-vasofrmative, spindled	Mass-forming, hypervascular
15	M	80	Mass-forming, vasofromative	Mass-forming, no hypervascularity
16	F	70	Mass-forming, vasofromative	Mass-forming, hypervascular
17	M	73	Mass-forming, vasofromative	Mass-forming, hypervascular
